# Leptin a potential link between obesity and depression

**DOI:** 10.1007/s00018-025-05892-6

**Published:** 2025-10-25

**Authors:** Lourdes Hontecilla-Prieto, Daniel José García-Domínguez, Carmen Berlanga-Gil, Rocío Flores-Campos, Raquel Muñoz-Pacheco, María D. Franco-Fernández, Juan A. Flores-Cordero, Flora Sánchez-Jiménez, Antonio Pérez-Pérez, Teresa Vilariño-García, Víctor Sánchez-Margalet

**Affiliations:** 1 Clinical Biochemistry Service, Virgen Macarena University Hospital, University of Seville, Seville, Spain; 2 Department of Medical Biochemistry and Molecular Biology and Immunology, School of Medicine, University of Seville, Seville, Spain; 3 Institute of Biomedicine of Seville, Virgen Macarena/Virgen Rocio University Hospital, CSIC, University of Seville, Seville, Spain; 4 Psychiatry Service, Virgen Macarena University Hospital, University of Seville, Seville, Spain; 5 Department of Medical Biochemistry and Molecular Biology and Immunology, Medical School, Virgen Macarena University Hospital,, University of Seville, Av. Sánchez Pizjuan 4, Seville, 41009 Spain

**Keywords:** Leptin, Obesity, Bipolar and depression disorders, Inflammation

## Abstract

Obesity poses a serious threat to public health, acting as an epidemic that affects individuals of all ages, ethnicities, and sociocultural backgrounds. Besides, psychiatric disorders, especially depression, are also highly prevalent. Given the high impact of both types of conditions and their potential interrelation, they have become the focus of numerous research endeavors. In fact, the relationship between obesity and depression is often viewed as bidirectional. Leptin, a hormone released by adipose tissue that regulates energy balance and appetite, is closely linked to obesity. Recent research indicates that this hormone may also influence mood and behavior, which motivates the analysis of leptin’s role in the shared pathophysiology between obesity and psychiatric disorders. The emerging relationship between obesity and neurodegeneration, attributed to the presence of chronic inflammation associated with this disease, prompts exploration of the potential role of leptin in this persistent inflammation, thus contributing to the neuroinflammatory state implicated in the genesis or evolution of depression. Therefore, in the present study, we aim to conduct a scoping review of the relationship between obesity and leptin with depression. According to the literature, there is an increase in leptin levels in depression, playing a pathophysiological role in the inflammation associated with the risk of depression in obesity. Nevertheless, further clinical studies are needed to fully understand the mechanisms and implications of leptin in the development of depression in obesity.

## Introduction

Depression is classified into two main diagnostic categories. According to the Diagnostic and Statistical Manual of Mental Disorders, Fifth Edition (DSM-5), published by the American Psychiatric Association, these are bipolar and related disorders—characterized by alternating episodes of depression and mania or hypomania—and depressive disorders, whose most representative form is major depressive episode, occurring without manic symptoms [1]. Alternatively, the World Health Organization’s International Classification of Diseases, 11th Revision (ICD-11), also recognizes two major diagnostic categories: bipolar disorders and depressive disorders, each including multiple subcategories [2].

The etiology of major depressive disorder is multifactorial, involving both genetic, biological, psychological and socio-environmental factors [3, 4]. A stronger association with genetic factors has been observed in cases where the onset of the disorder occurs at an early age [5, 6]. In addition, several risk factors for the development of this disorder have been identified in elderly patients, including neurodegenerative diseases such as Alzheimer’s and Parkinson’s, stroke, multiple sclerosis, epilepsy, cancer, macular degeneration, and chronic pain [7, 8]. Moreover, certain traumatic events can also act as triggers for depression [9]. Recently, the concept of “immunometabolic depression” (IMD) has been proposed as a biologically more homogeneous subtype within the spectrum of major depression. This phenotype is characterized by the presence of atypical depressive symptoms related to energy metabolism, such as hypersomnia, hyperphagia, fatigue, anhedonia and leaden paralysis, in combination with low-grade systemic inflammation and metabolic disturbances such as obesity, dyslipidemia, and insulin and leptin resistance. At the genetic and epidemiological level, individuals with IMD have a higher burden of variants associated with immuno-metabolic biomarkers such as leptin, triglycerides and body mass index (BMI), suggesting a bidirectional causality between these factors and the onset of depression. Clinically, it has been observed that patients with IMD show a lower response to conventional antidepressants, while therapeutic strategies targeting metabolism or inflammation (such as statins, pioglitazone, GLP-1 agonists, anti-inflammatory drugs or lifestyle interventions) seem to be more effective [10].

Obesity, a metabolic disorder, is described as a risk factor for depression. Both conditions are significant public health issues due to their high prevalence and elevated rates of morbidity and mortality. Recent scientific evidence suggests that depression and obesity are interrelated in a dependent manner. Specifically, depression and obesity exhibit a bidirectional relationship [11, 12], in which each condition can act both as a contributing factor to and a consequence of the other [13]. In fact, obesity and depression are highly prevalent diseases worldwide and have been recognized as public health priorities [14].

Obesity, a condition marked by excessive adipose tissue accumulation and dysregulation of the balance between appetite control and energy expenditure [15, 16], is characterized by excessive accumulation of adipose tissue, which results in adipose tissue dysfunction. This dysfunction is marked by adipocyte hypertrophy and inflammation, leading to altered secretion of adipokines such as leptin. Therefore, leptin has been recognized as playing a pivotal role in the pathogenesis of obesity. Leptin is a protein hormone primarily synthesized in white adipose tissue and is involved in the regulation of body weight [17]. This hormone acts as a satiety signal in the brain, with a particular focus on the hypothalamus, where it regulates food intake and energy metabolism [18]. Commonly referred to as the “satiety hormone,” leptin serves as a link between an individual’s nutritional status and the neuroendocrine and immune systems [19]. Additionally, leptin not only contributes to the control of food intake, but also plays a role in various brain functions, including neuronal development and survival, as well as aspects of behavior and cognition [20]. Therefore, the alteration in its levels may contribute to the emergence of psychopathological symptoms [21]. It is known that disruption of leptin signaling can impair the regulation of the adipo-insular axis, which links adipose tissue, the central nervous system, and the endocrine system, affecting appetite control, mood, and cognitive function [21]. In addition, in situations of leptin resistance such as those induced by high-fat diets, functional and structural alterations in the hippocampus have been demonstrated that correlate with an impairment in executive functions, reward processing and inhibitory control, phenomena that have also been documented in humans with obesity and cognitive disorders [22].

Leptin has been investigated as a potential biomarker of stress, as its levels have decrease during acute stress episodes [23]. Stress can cause anxiety, depression, increased risk of suicide, as well as elevated cardiovascular risk, disordered eating behaviors, and obesity. One of the effects of leptin, as previously mentioned, is to promote the inflammatory process, activating monocytes and lymphocytes [24, 25]. The study of inflammation in depression is an emerging field, increasingly recognized as a mechanism that may contribute to the pathogenesis of clinical depression [26–29]. It has been described that proinflammatory cytokines induce neuroinflammation and influence behavior and emotional regulation [30] Indeed, there is substantial evidence that individuals with depression exhibit elevated levels of inflammatory markers in the blood, indicating the role of inflammation in the pathophysiology of depression [26]. The emerging relationship prompts exploration of the potential role of leptin in this persistent inflammation, thus contributing to the neuroinflammatory state implicated in the genesis or evolution of depression.

**Fig. 1:**
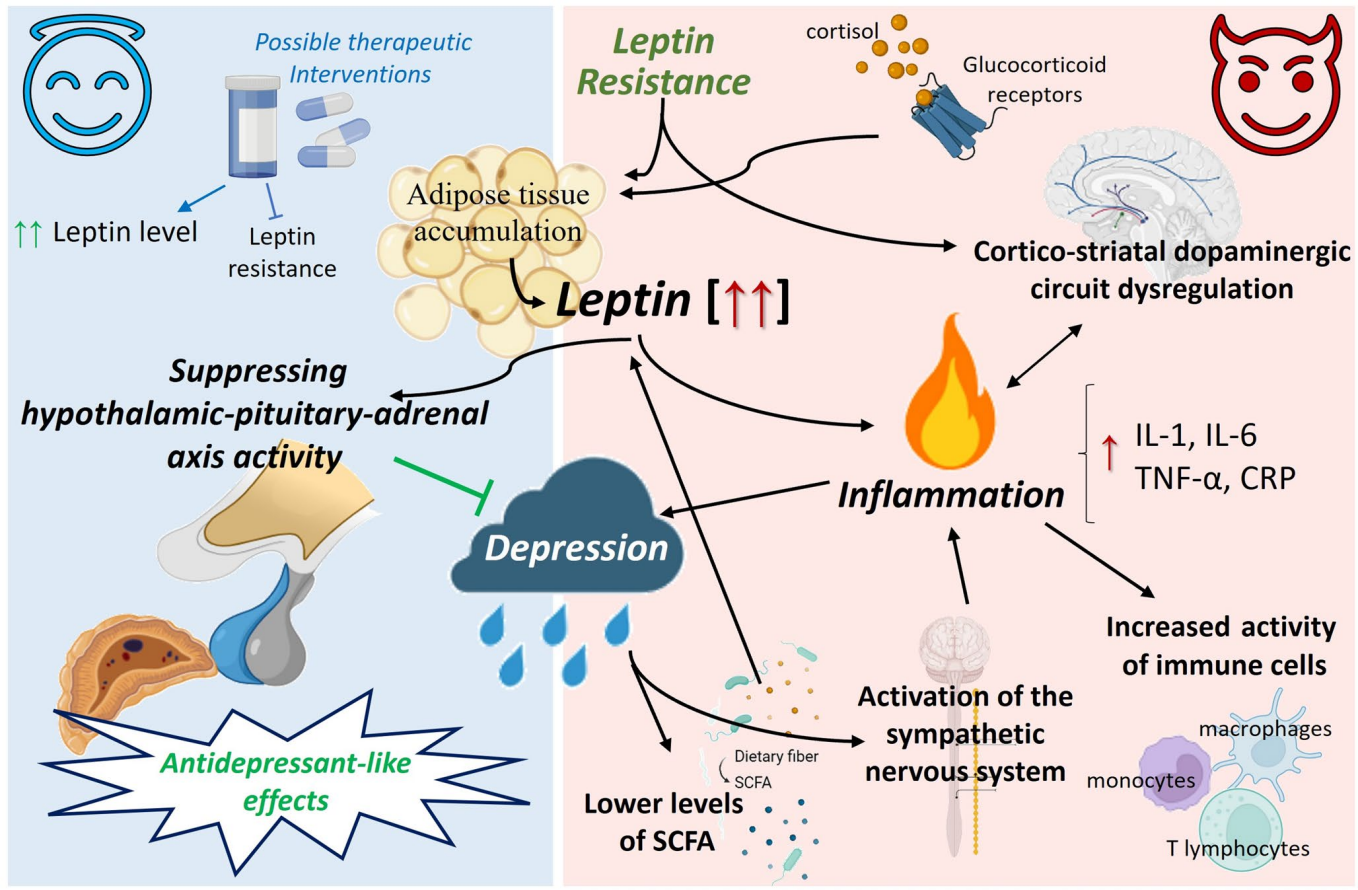
The vicious circle of obesity and depression. Central pro-depressive role of leptin in this positive feedback loop. The central pro-depressant role of leptin in this positive feedback loop is highlighted. Eating disorders, obesity-associated metabolic syndrome and leptin resistance lead to an accumulation of adipose tissue, which in turn increases leptin concentrations. Leptin, among other processes, promotes inflammation, which contributes to the development of depression and bipolar disorder. Furthermore, the hypothalamic-pituitary-adrenal axis and leptin resistance are pointed out as possible therapeutic targets to disrupt the described feedback mechanism

Overall, this review provides a comprehensive overview of the relationship between depressive disorders, obesity, and leptin, enhancing the understanding of their interconnections (Fig. 1). Additionally, it explores the association between leptin and inflammation in patients with depression.

## Bipolar disorder

### Obesity and bipolar disorder

The prevalence of obesity among patients with bipolar disorder is nearly twice has been consistently reported across different populations, suggesting a global trend rather than a geographically isolated phenomenon [31]. For instance, in Canada, the prevalence of obesity in bipolar patients is higher with rates of 42.3% compared to 24.1% in healthy individuals. Similarly, the prevalence of metabolic syndrome is higher in bipolar patients (35.7%) than in healthy controls (19.1%) [32]. These metabolic disturbances have been attributed to multiple interacting factors, including the use of drugs, many of which are associated with weight gain and metabolic alterations, as well as lifestyle-related aspects such a low physical activity and poor diet [33–35]. That increased prevalence does not appear to be associated with lithium treatment, as similar rates were observed in patients receiving lithium and those who were not. However, further studies with larger sample sizes are needed to confirm this association [32]. Other studies have also reported a higher rate of obesity in individuals with bipolar disorder compared to the General population, with prevalence estimates ranging from 40 to 60% among affected patients [36].

Obesity and bipolar disorder share a complex pathophysiology involving genetic, neurobiological, and environmental factors. Among the common mechanisms are chronic inflammation, oxidative stress, mitochondrial dysfunction, and insulin resistance. Both conditions also exhibit alterations in hunger regulation, glucose metabolism, hypothalamic-pituitary-adrenal (HPA) axis activity, and neurotransmission—particularly involving serotonin and dopamine. These overlapping pathophysiological features may help explain the high comorbidity observed between the two disorders [37]. In this context, individuals with bipolar disorder are more Likely to adopt unhealthy dietary habits and lead sedentary Lifestyles, which contributes to the increased prevalence of obesity. Moreover, bipolar patients are more prone to stress, anxiety, and depression, all of which are factors that may increase the risk of developing obesity. In a study of 139 patients with bipolar disorder and 93 controls, the relationship between food cravings, hormone levels (including leptin), psychotropic medication and clinical status was analyzed. It was observed that people with bipolar disorder had a higher frequency of total food cravings, fat cravings and fast food cravings than the control group of healthy people and, in addition, more frequent fast food cravings were associated with more depressive symptoms [38]. In addition, the use of atypical antipsychotics, commonly prescribed in the treatment of bipolar disorder, is associated with weight gain and increased BMI [37, 39]. In this context, patients with bipolar disorder with a particularly high risk of weight gain are treated with second-generation antipsychotics (olanzapine, quetiapine), which implies a higher frequency of craving for sweet foods than those who were not taking this type of medication [38].

### Leptin and bipolar disorder

A systematic review of 32 studies investigated the role of appetite-regulating hormones in bipolar disorder (Table 1). It found that half of them reported elevated leptin levels, particularly when patients with bipolar disorder were analyzed as a single group without subdivision by mood state. The meta-analysis yielded the following results: K = 21, g = 0.36, 95% CI: 0.14–0.58, *p* = 0.003, I² = 79.9%. When stratified by mood phase, the highest leptin levels were observed in patients in a euthymic state: K = 10, g = 0.35, 95% CI: 0.03–0.68, *p* = 0.036, I² = 74.2% [40]. However, after excluding specific individual studies, these differences were no longer statistically significant. No significant differences in leptin levels were found in patients during manic episodes (K = 3, g = 0.41, 95% CI: − 1.08 to 0.89, *p* = 0.360, I² = 82.4%) or depressive episodes (K = 4, g = 0.12, 95% CI: − 0.95 to 1.19, *p* = 0.739, I² = 88.2%) [40]. Furthermore, a meta-regression analysis in this study found that higher-quality studies were associated with larger estimated differences in leptin levels between groups. It was also concluded that the biological material used for leptin measurement significantly influenced the results. Specifically, studies using plasma samples provided more accurate effect size estimates, indicating that plasma is a more reliable medium for leptin quantification [40].

**Table 1:** Leptin results in bipolar and depression disorders

	Study and population sample	Key Findings	Ref.
***Leptin and Bipolar Disorder***	A systematic review of 32 studies	The highest leptin levels were observed in patients in a euthymic state: K = 10, g = 0.35, 95% CI: 0.03–0.68, *p* = 0.036, I² = 74.2%.	40
		No significant differences in leptin levels were found in patients during manic episodes (K = 3, g = 0.41, 95% CI: − 1.08 to 0.89, *p* = 0.360, I² = 82.4%) or depressive episodes (K = 4, g = 0.12, 95% CI: − 0.95 to 1.19, *p* = 0.739, I² = 88.2%)	40
		A meta-regression analysis found that higher-quality studies were associated with larger estimated differences in leptin levels between groups.	40
	28 bipolar disorder type I patients, 36 bipolar disorder type II patients and 66 healthy controls	Not significant differences in leptin levels between bipolar patients and healthy control.	41
		Positive correlation between leptin and C-reactive protein levels in healthy controls and patients with bipolar disorder type II.	41
	A systematic review and meta-analysis	Increase leptin levels together with elevated BMI and age	42
***Leptin and Depression***	Depressed patients (*n* = 24) and a healthy control group (*n* = 33)	Leptin levels in patients with depression and healthy controls are similar	58
	15 patients with depression (8 women, 7 men) and 15 age- and sex-matched controls (8 women and 7 men)	Depression was associated with higher serum leptin compared to control subjects.	59
	12 female and 8 male patients with major depression, and 12 female and 8 male normal controls	Baseline plasma leptin concentrations were significantly higher in the female patients compared to the female controls.	60
	69 patients who suffered from major depressive disorder and 51 healthy controls	Leptin levels were low in patients with major depressive disorder.	61
	31 males and 36 females	Women with depression demonstrated the existence of a significant correlation between the severity of depressive symptoms and various measures of obesity, which proved to be an independent predictor of depression severity in regression analyses (β = 0.60, *p* = 0.007), explaining up to 34% of the variance.	64
	537 patients with depression (minimal to no depression *n* = 415; mild depression *n* = 63; moderate to severe depression *n* = 59)	Patients with moderate to severe depression exhibited significantly higher plasma leptin concentrations (median: 37.7 ng/mL; interquartile range [IQR]: 17.6–64.9), compared to those with mild depression (22.9 ng/mL [IQR: 7.0–57.9]) or minimal/no symptoms (19.8 ng/mL [IQR: 7.8–39.1]; *p* = 0.003).	57

Conversely, another study with 28 bipolar disorder type I patients, 36 bipolar disorder type II patients and 66 healthy controls, did not find significant differences in leptin levels between bipolar patients and healthy controls. However, a positive correlation between leptin and C-reactive protein (CRP) levels was observed in both healthy controls and patients with bipolar disorder type II, but not in those with bipolar disorder type I. Based on these findings, the authors suggested that elevated CRP levels in bipolar disorder type I may contribute to leptin resistance or typing in bipolar disorders. Furthermore, this dysregulation may be due to obesogenic diets, medications and/or Alzheimer’s disease per se. Finally, the possible additive or even synergistic effects should be taken into account, as both leptin and CRP could simultaneously increase in bipolar disorder [41] (Table 1).

### Leptin, obesity and bipolar disorder: their implications in inflammation

Leptin is currently under investigation as a potential biomarker in the pathophysiology of bipolar disorder. Given the relationship between leptin and inflammation, this association has been studied in patients with bipolar disorder. According to some studies, the disproportionate increase in leptin levels together with elevated BMI and age of these patients would indicate that such adipose tissue is potentially inflammatory and leptin would have a role as a biomarker of neuroprogression in bipolar disorder [42] (Table 1). Moreover, a significant relationship has been identified between leptin and the immunological parameter sTNF-R1 (soluble tumor necrosis factor receptor 1), which is released during systemic inflammation—though this association was specifically noted during the full remission phase of the disorder with normal or over body weight. This finding indicates that symptomatic bipolar patients do not have the same pathway regulating body adiposity as euthymic patients or normal adults and leptin may play a role in modulating the immune response and inflammation in bipolar disorder [43]. It is described that elevated leptin levels in patients during the euthymic phase may exert a protective effect by suppressing the HPA axis. Since the HPA axis is typically overactivated in patients with bipolar disorder [44], leptin’s suppressive action could confer physiological benefits by mitigating this dysregulation [45].

On the other hand, active inflammatory markers have been identified during the acute manic phase, as evidenced by elevated levels of interleukin-1 receptor antagonist (IL-1Ra), high-sensitivity C-reactive protein (hs-CRP), and soluble tumor necrosis factor receptor 1 (sTNF-R1). Moreover, patients with bipolar disorder may exhibit a state of chronic inflammation, characterized by increased IL-1Ra and hs-CRP levels during acute mania, along with sustained elevations in hs-CRP even during full remission [46]. Leptin acts as a modulator of inflammatory processes. It promotes the production of pro-inflammatory cytokines and regulates both innate and adaptive immune responses [47, 48]. Leptin also influences the synthesis of CRP, an acute-phase protein produced by hepatic and endothelial cells in response to inflammation. Studies have shown a positive correlation between circulating leptin levels and CRP concentrations [41]. Moreover, the inflammation mediated by excess leptin in obesity is known to participate in the leptin resistance in the central nervous system, leading to a further increase in body weight, producing a positive feed-back that perpetuates obesity [49]. It can be inferred that leptin plays a central role in the acute manic phase by promoting inflammation and their underlying mechanisms.

Alterations in central neural networks can lead to impaired reward processing, a phenomenon observed in bipolar disorder, which has also been associated with systemic inflammation. This inflammatory state is thought to induce changes in the connectivity of the cortico-striatal dopaminergic circuit. Furthermore, some studies have demonstrated a correlation between bipolar disorder and BMI, both of which are associated with similar patterns of regional brain volumes [41]. Moreover, a negative correlation has been observed between leptin levels and functional connectivity within the cortico-striatal circuit. The interaction between affective reward circuits and interoceptive networks may help explain the heightened vulnerability to metabolic syndrome in individuals with bipolar disorder. Changes in BMI and insulin sensitivity are associated with the intrinsic functional organization of the brain. In patients with bipolar disorder, central leptin resistance appears to play a key role in the dysregulation of cortico-striatal pathways, influencing food choices and increasing the likelihood of higher BMI in this population [41]. Finally, leptin serves as a key mediator in the communication between the brain and adipose tissue, maintaining energy homeostasis and regulating motivation through neural reward circuits [50].

## Depression

### Obesity and depression

Obesity and depression are closely linked with a bidirectional relationship where each pathology can increase the risk of developing the other. Thus, obese people are more likely to suffer from depression, and those who suffer from depression are more likely to be obese. According to three cross-sectional studies, a pooled odds ratio (OR) of 1.33 was observed between the two disorders [51]. Additionally, a meta-analysis of longitudinal studies in adolescent populations reported similar ORs for the development of depression in individuals with obesity (OR: 1.4) and for the development of obesity in individuals with depression (OR: 1.7), highlighting the bidirectional nature of this relationship [51]. A stronger association has been documented among female patients, particularly adolescent girls, which has been attributed to the earlier onset of puberty and the associated biological and hormonal changes [52, 53]. In a multinational study, a higher prevalence of obesity was observed among women with depression, especially among South American, Caucasian, and Asian women [54].

Furthermore, a meta-analysis exploring the relationship between metabolic syndrome and the onset of depression found a significant positive association when examining individual parameters, such as central obesity or hypertriglyceridemia, with an OR of 1.20 (95% CI: 1.07–1.35) [55]. The same meta-analysis also investigated the risk of developing obesity in patients with depression and identified a significant association, with an OR of 1.31 (95% CI: 0.99–1.73), further supporting the bidirectional link between these two conditions [55]. Other studies have reported similar findings. For example, among obese individuals, the OR for developing depression was 1.55 (95% CI: 1.22–1.98; *p* < 0.001), while for individuals with depression, the OR for developing obesity was 1.58 (95% CI: 1.33–1.87; *p* < 0.001) [56].

Finally, a clear association has been established between depression, obesity, and their related complications. For instance, one study reported a 50% higher prevalence of metabolic syndrome in individuals with depression and a fourfold increased risk of developing this syndrome following a major depressive episode. Similarly, individuals with moderate to severe depression had higher BMI values compared to those with mild depression (33 ± 8 vs. 31 ± 9 vs. 29 ± 7 kg/m², *p* < 0.001) [57].

### Leptin and depression

Given the demonstrated bidirectional relationship between depression and obesity, several studies have explored the relationship between leptin levels and major depressive disorder, yielding heterogeneous results. There are studies indicating that leptin levels in patients with depression and healthy controls are similar [58], while other studies show that an association in these patients with increased [59, 60] or decreased leptin levels [61–63] (Table 1). The heterogeneity of the results may be due to confounding factors that have not been taken into account in the studies. Among them, differences between males and females in the association of depression with obesity may contribute to such inconsistency. One study demonstrated a significant association between elevated leptin levels and depression only in female patients, where gender differences are important in the association between clinical parameters, highlighting that elevated leptin levels appear to be a potential mechanism linking depression to obesity in women. In this same study women with depression demonstrated the existence of a significant correlation between the severity of depressive symptoms and various measures of obesity, which proved to be an independent predictor of depression severity in regression analyses (β = 0.60, *p* = 0.007), explaining up to 34% of the variance [64] (Table 1). Moreover, such an association between leptin and depression in women was observed both before and after treatment with antidepressants [64]. This suggests a potential interaction between leptin, sex, and treatment response in the context of depression [65]. Additionally, it has been proposed that the link between leptin and depression may be mediated by increased adiposity in individuals with more severe depressive symptoms. In this regard, patients with moderate to severe depression exhibited significantly higher plasma leptin concentrations (median: 37.7 ng/mL; interquartile range [IQR]: 17.6–64.9), compared to those with mild depression (22.9 ng/mL [IQR: 7.0–57.9]) or minimal/no symptoms (19.8 ng/mL [IQR: 7.8–39.1]; *p* = 0.003) [57] (Table 1). Furthermore, when using the Beck Depression Inventory-II (BDI-II), a quantitative association was identified: for every one-point increase in the BDI-II score, plasma leptin levels increased by 17%, further supporting the hypothesis of a proportional relationship between the severity of depressive symptoms and leptin concentrations [57]. These findings suggest a complex and potentially bidirectional relationship between leptin, obesity, and depression, modulated by clinical and demographic variables such as sex and body mass index.

Finally, based on findings of decreased leptin levels or resistance in patients with depression a “leptin hypothesis of depression” has emerged [66]. Chronic stress acts as a predisposing and contributing factor to the onset of depression in humans. Thus, the induction of chronic stress in rats or mice causes them to develop behavioral deficits and endocrine abnormalities, which mimic the symptoms of human depression. Rats exposed to chronic stress showed a decrease in basal plasma leptin levels independent of body weight [67]. These findings raised the hypothesis that leptin insufficiency might underlie depression-like behavior. Supporting this theory, pharmacological experiments have shown that leptin produces antidepressant-like effects in the Porsolt forced swim test, a standard behavioral assay for assessing depression-like states in animal models [67, 68]. Thus, systemic administration of leptin may reverse chronic stress. Although it appears that leptin may act as an antidepressant, the evidence for a causal relationship between depression and leptin insufficiency remains weak.

### Leptin, obesity and depression: their implications in inflammation

There is strong clinical and preclinical evidence supporting a link between inflammation and depression, as elevated levels of proinflammatory cytokines, including IL-1, IL-6, TNF-α and CRP, have been consistently observed in patients with depression [69]. Similar increases in inflammatory markers are also observed in rodents subjected to olfactory bulbectomy, a validated experimental model for inducing depressive-like behavior [70]. Moreover, elevated IL-6 and CRP levels have been shown to predict the onset of depressive symptoms, and vice versa [71]. In addition, other neuropsychiatric pathologies related to inflammatory dysfunction have also shown significant associations between leptin and affective symptoms. In a study conducted in patients with alcohol use disorder (AUD), significantly higher levels of plasma leptin and inflammatory markers (IL-6, CRP, TNF-α) were observed after one month of abstinence compared to healthy controls. Leptin levels showed a positive correlation with inflammatory markers [26, 72].

Human studies further corroborate the role of inflammation in depression. In patients treated with interferon-alpha (IFN-α) for hepatitis C or cancer, up to 50% developed depressive symptoms such as anxiety, pain, anhedonia, fatigue, cognitive impairment, sleep disturbances, irritability, hostility, appetite loss, and suicidal ideation. These findings strongly suggest that cytokines elevated by IFN-α therapy contribute directly to the development of depressive symptoms [1].

Depression is also associated with activation of the sympathetic nervous system (SNS), which may suppress antiviral immune responses and increase susceptibility to infections [71]. Simultaneously, SNS activation enhances pro-inflammatory activity, aimed at fighting bacterial and other extracellular pathogens. This dysregulation between inflammatory and antiviral immune responses is thought to play a significant role in the pathophysiology of depression. Immune activation in depression is evidenced by elevated markers of inflammation and increased activity of immune cells such as macrophages, monocytes, and T lymphocytes [73].

Finally, the role of leptin in the inflammatory process associated with depression has been examined. A meta-analysis suggests that the relationship between leptin and depression may be modulated by factors such as age and BMI. While no statistically significant difference in leptin levels was observed between patients with major depressive disorder and healthy controls (95% CI: − 0.06 to 0.31; *p* = 0.170), subgroup analyses revealed higher leptin levels in older individuals (≥ 40 years) with a BMI ≥ 25 who had major depressive disorder compared to controls [74].

Leptin, obesity and depression are interconnected, and inflammation may act as a key link. Obesity can lead to increased leptin levels and inflammation, which may contribute to depressive symptoms. On the other hand, depression may also be associated with changes in leptin levels and the inflammatory process, making their relationship even more complicated. As previously described, increasing evidence suggests that the chronic inflammatory state induced by excess adipose tissue plays a key role in the pathogenesis of psychiatric disorders. The inflammatory model and cytokines such as leptin could generate an imbalance in the immune system, characterized by elevated levels of proinflammatory cytokines or reduced production of anti-inflammatory cytokines, which would be a critical factor in the development of clinical depression [75].

## Final remarks

Bipolar disorder is frequently associated with comorbid conditions and increased mortality, resulting in a Life expectancy up to 12 years shorter than that of the general population [40]. For instance, patients with bipolar disorder show higher rates of obesity and type 2 diabetes mellitus compared to age- and sex-matched controls [40]. The association between bipolar disorder and obesity has been corroborated by several studies, including a meta-analysis of 37 articles, which confirmed a significantly higher prevalence of obesity among individuals with bipolar disorder [76]. This potential link gained further support from findings in the U.S. National Epidemiologic Survey, which demonstrated a positive correlation between both conditions [36].

These comorbidities may share common pathophysiological mechanisms. For instance, similarities have been observed in the reward system activation during binge eating episodes and hypomanic states. Neuroimaging studies have revealed that regulatory pathways—particularly those involving serotonin and dopamine—play a role in both energy homeostasis and mood regulation. Additionally, bipolar disorder and obesity are both associated with cognitive dysfunction and elevated BMI. These conditions are also negatively correlated with memory performance and executive function [77].

Moreover, the reward system activated following food intake is regulated by hormones such as leptin, which acts on dopaminergic neurons in the midbrain [78]. These hormones can be modulated by the gut microbiota and its metabolites, such as short-chain fatty acids (SCFAs) or lipopolysaccharides [79]. Besides, in patients with bipolar disorder, lower levels of SCFA-producing bacteria have been observed, which may result in altered leptin levels in these individuals [79]. Furthermore, appetite-regulating hormones influence the inflammatory immune response, which is known to be dysregulated in bipolar disorder [40].

The relationship between obesity and the course of depression has been linked to dysregulation of both inflammation and the HPA axis. In this context, cortisol, through glucocorticoid receptors in fat stores—particularly in abdominal adipose tissue—suppresses lipid-mobilizing enzymes [80]. Moreover, depression is frequently associated with unhealthy lifestyle habits, which also contribute to the development of obesity [56]. Along the same lines, early-onset obesity during the course of depression has been found to be more common in women than in men [81].

**Fig. 2:**
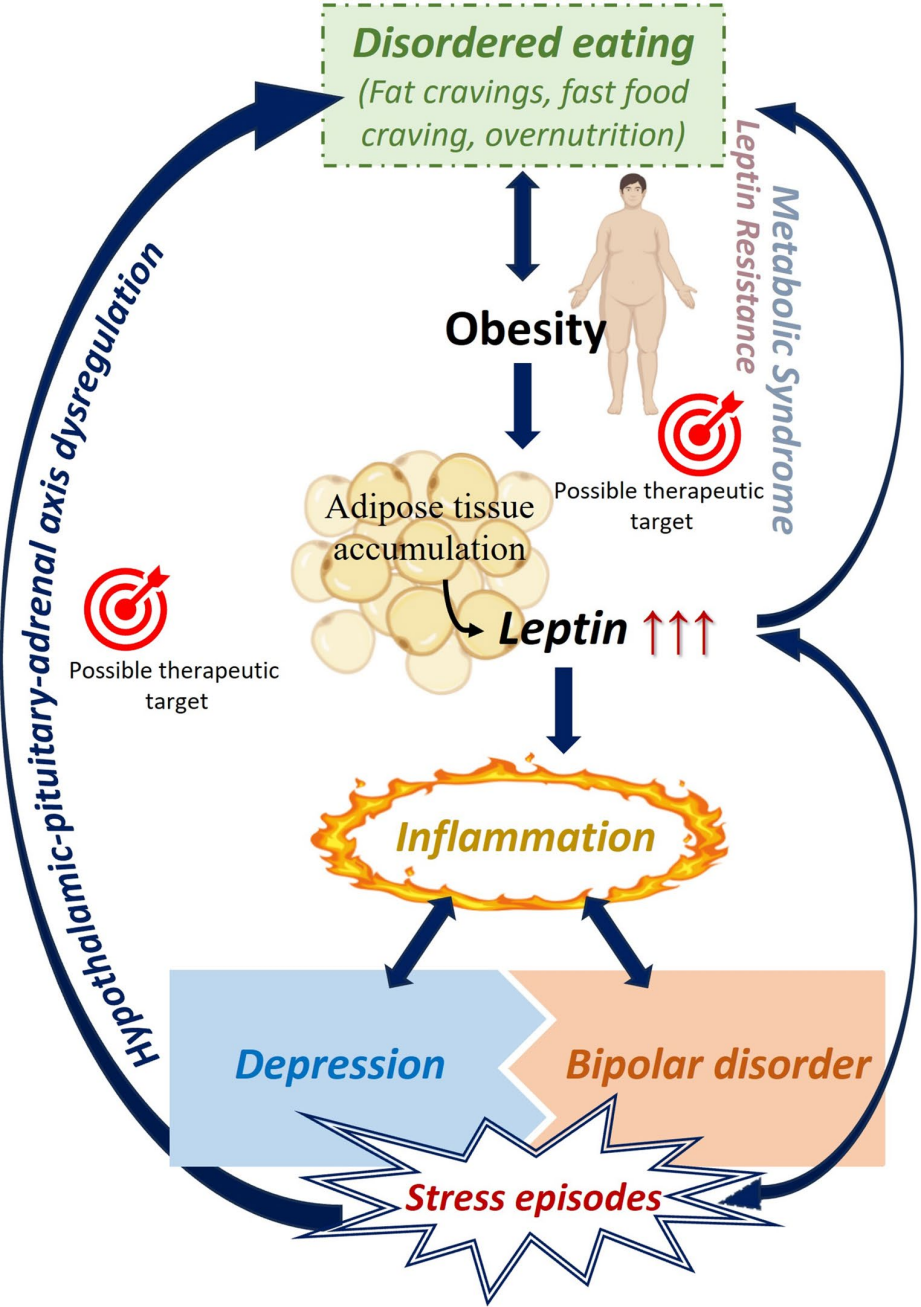
Pro- or anti-depressive role of leptin in the context of obese patients. Mechanistic relationship centered on the Leptin–Inflammation–Depression axis. On the left side of the image (blue background), the antidepressant role reported in relation to increased leptin concentration is highlighted, based on the central role of the hypothalamic-pituitary-adrenal axis. In the red background, the pro-depressant role of leptin is emphasized. Through increased inflammation and the release of associated regulatory molecules (IL-1, IL-6, etc.), depressive symptoms are promoted. In addition, the systems and organs involved in the reinforcement of this pro-depressant leptin-inflammation axis are detailed

Throughout this review, we have highlighted the significant pro-depressive impact of elevated leptin levels or leptin resistance in obese patients (Fig. 2). However, existing evidence suggests that patients with low leptin levels or leptin resistance tend to exhibit depressive symptoms. Leptin resistance could be treated with selective serotonin reuptake inhibitors, such as fluvoxamine, which restores leptin-induced STAT3 phosphorylation in ER stressed cells and potentiates the anorexigenic effects of leptin in ob/ob mice [82]. Also, it is important to know that other factors such as vitamin D deficiency could negatively modulate leptin signaling, contributing to the neuroendocrine imbalance characteristic of depressive disorders [63]. Preclinical studies in rodent models have shown that leptin administration exerts antidepressant-like effects [67], opening new perspectives for therapeutic interventions (Fig. 2). These could aim to improve leptin sensitivity as a strategy to simultaneously address obesity, associated metabolic syndrome, and depression.

In light of leptin’s seemingly contradictory roles, further studies are needed to fully elucidate the role of leptin in the pathophysiology of depression and to identify the modulatory factors that influence its pro- or anti-depressive effects.

## Data Availability

Not applicable.
